# Supplementary material for: Piceatannol Inhibits the Immunostimulatory Functions of Dendritic Cells and Alleviates Experimental Arthritis
Source: Int J Mol Sci. 2025 Apr 11;26(8):3626. doi: 10.3390/ijms26083626 (PMC12026827; doi:10.3390/ijms26083626)
Supplement: Supplementary file 1 [file ijms-26-03626-s001.zip › ijms-3473583-supplementary.pdf]

Piceatannol inhibits the immunostimulatory functions of dendritic cells and alleviates experimental arthritis

Luyang Han[1, a], Peng Han[1, a], Yanbo Zhu[a], Jiawei Dong[a], Zhenyang Guan[a], Yuekang Xu [a], Jinyao Li [a, *] and Xiaoying Liu [a, *]

a. Xinjiang Key Laboratory of Biological Resources and Genetic Engineering, College of Life Science and Technology, Xinjiang University, Urumqi 830017, China

1 The two authors contribute equally.

* Correspondence:

Jinyao Li, College of Life Science and Technology, Xinjiang University, No. 777, Hua Rui Road, Urumqi 830017, China.

Email: ljyxju@xju.edu.cn

Xiaoying Liu, College of Life Science and Technology, Xinjiang University, No. 777, Hua Rui Road, Urumqi 830017, China.

Email: liuxy@xju.edu.cn

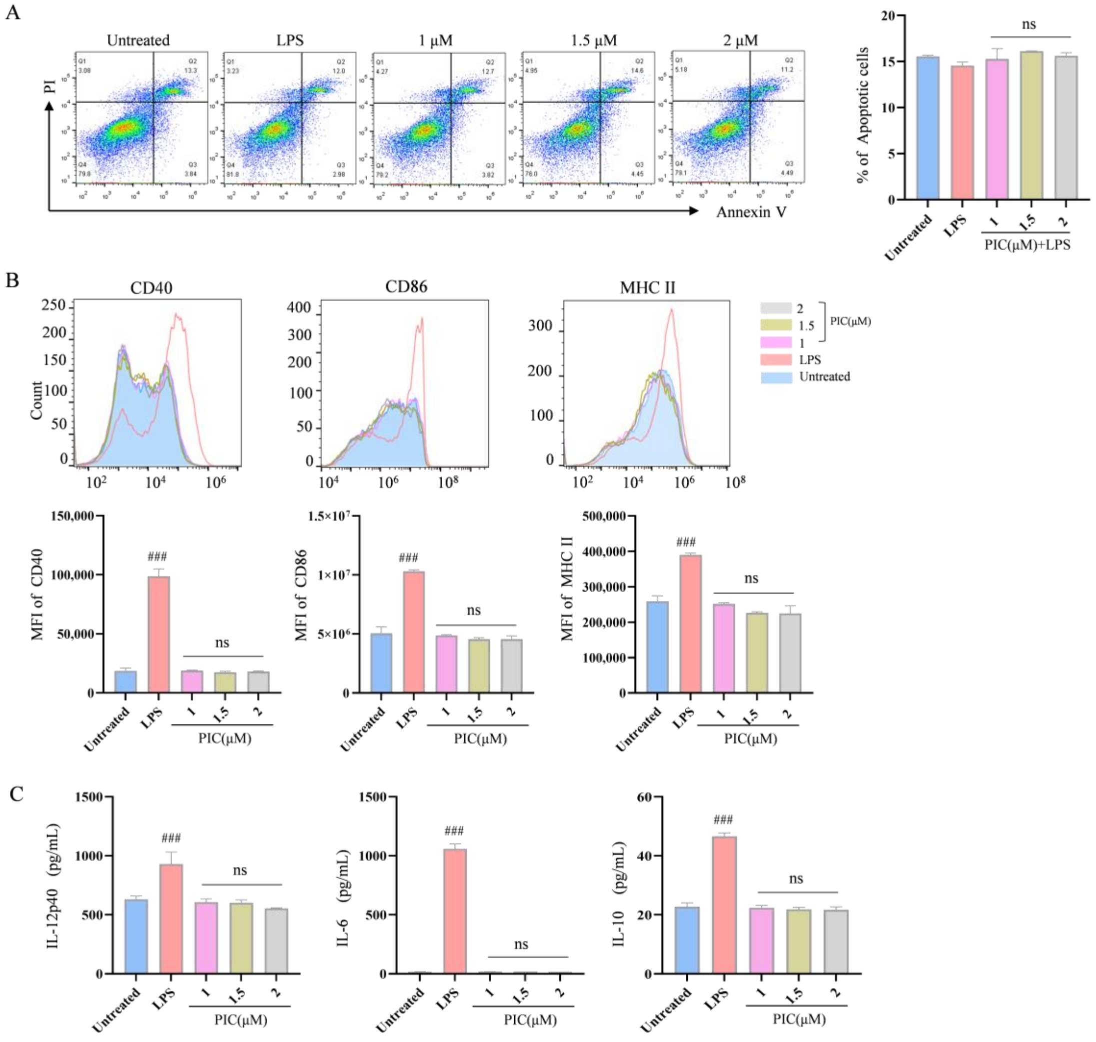

Figure S1. The effect of PIC on the maturation of DCs. Flow cytometry was used to detect the effect of the effective concentration of the drug on apoptosis and the effect on the maturation and secretion of cytokines of normal DCs; (A) There was no effect on the apoptosis of DCs at the effective concentration of PIC; (B) DCs treated solely with PIC exhibited no changes in the expression of surface molecules CD40 and CD86; (C) There was no significant change in the cytokines secreted by DCs after PIC treatment alone. ### P < 0.001 compared to the untreated group. “ns” compared to Untreated. ns: no significant.

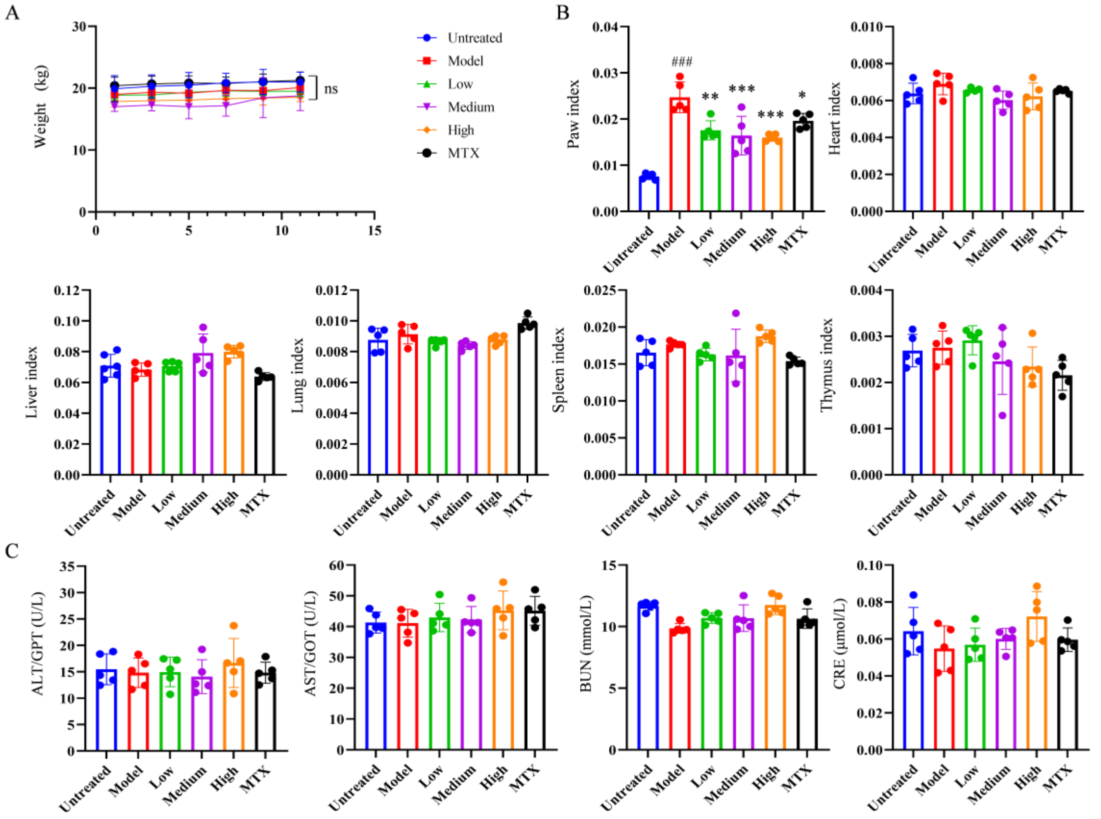

Figure S2. PIC treated CFA-induced arthritis in mice. The toxicity of the drug was evaluated by body weight, organ index, liver and kidney function in mice; (A) Changes in body weight of mice during modeling and treatment; (B) The organ index of mice is the ratio of the organ weight of mice to the last weight; (C) Serum was collected to detect liver and kidney function in mice; ### $P < 0.001$ compared to untreated group. * $P < 0.05$, ** $P < 0.01$ and *** $P < 0.001$, compared to the LPS group.

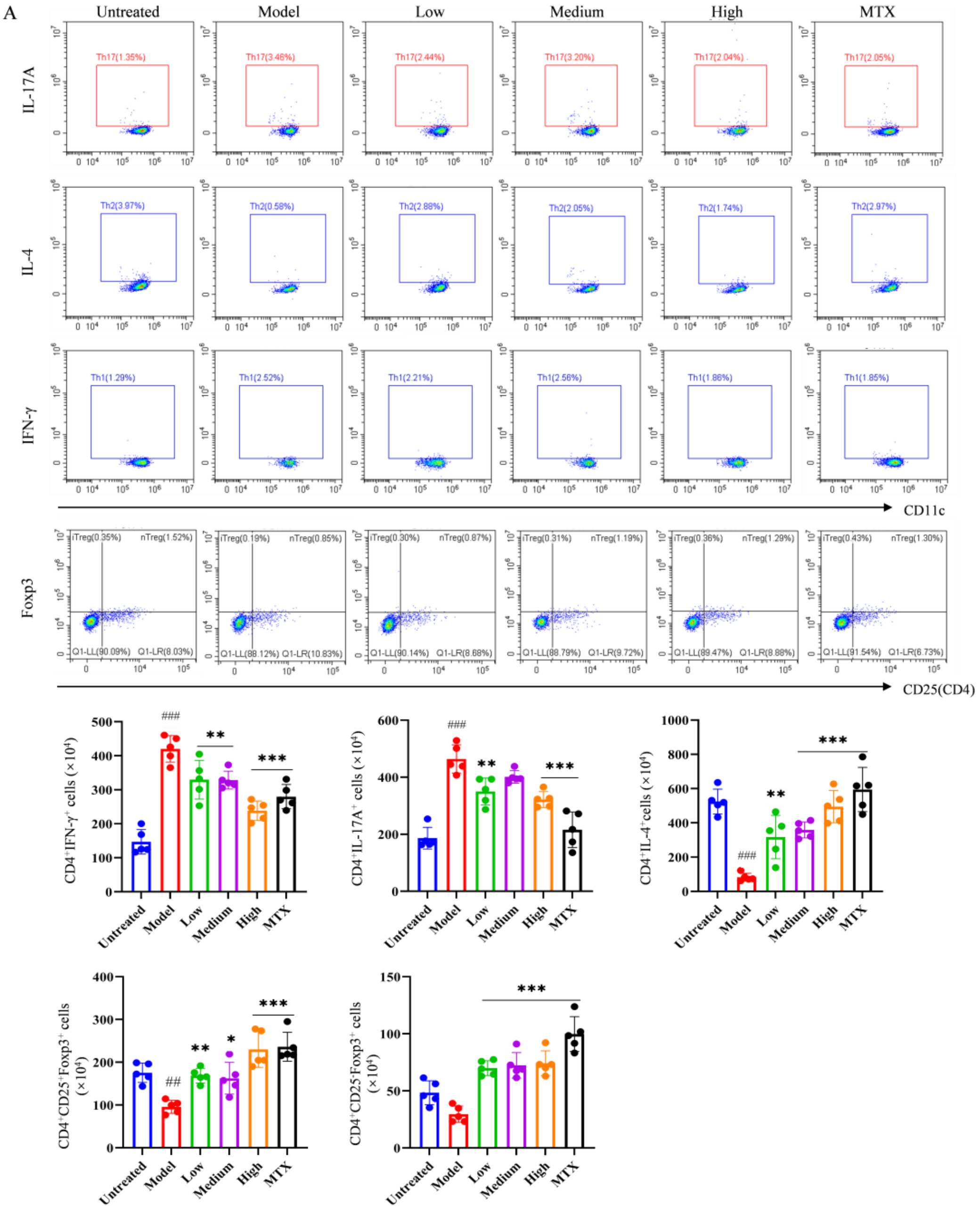
A
Untreated
Model
Low
Medium
High
MTX
IL-17A
IL-4
IFN-γ
CD11c
Foxp3
CD25(CD4)
CD4+IFN-γ+ cells (×10⁴)
CD4+IL-17A+ cells (×10⁴)
CD4+IL-4+cells (×10⁴)
CD4+CD25+Foxp3+ cells (×10⁴)
CD4+CD25-Foxp3+ cells (×10⁴)

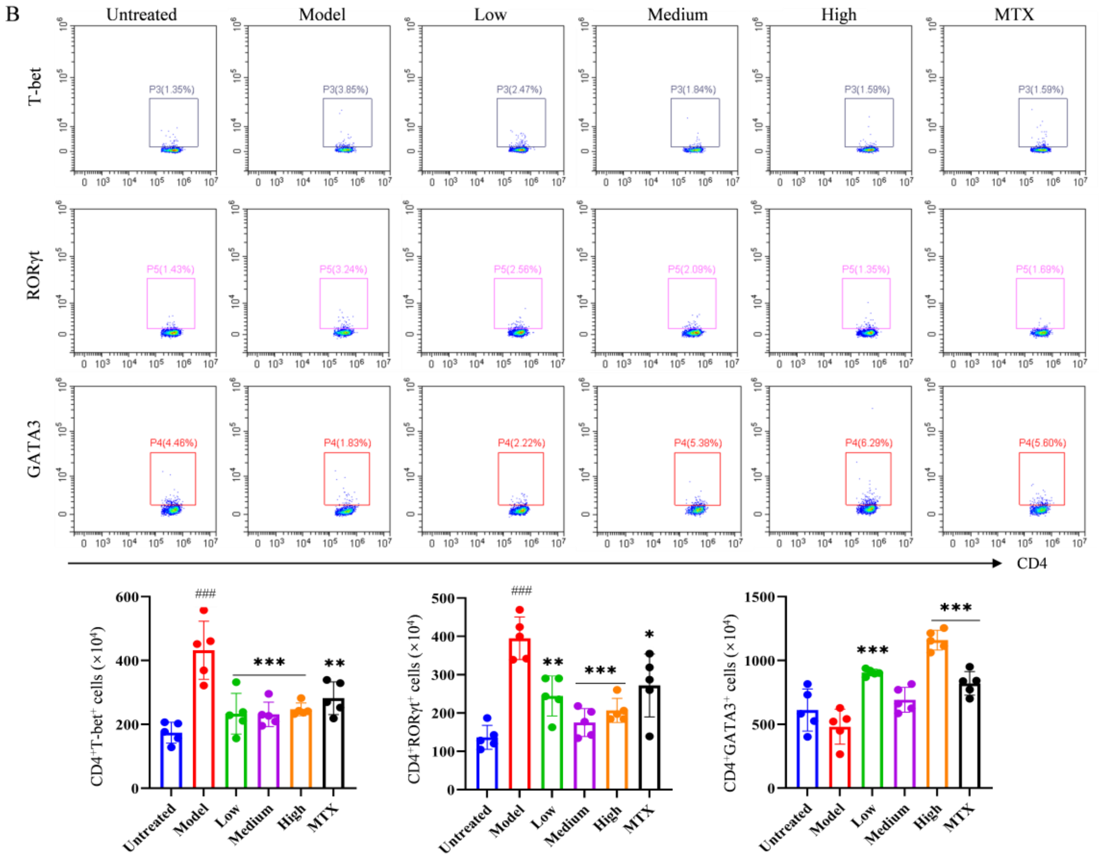

Figure S3. The effect of PIC on immune cells in spleen of arthritic mouse model. (A) Different cytokines secreted by Th1, Th2 and Th17 cells in the spleen of mice were detected by flow cytometry; (B) The differentiation of spleen T cells in model mice was verified by detecting nuclear transcription factors. $^{\#\#}$ $P < 0.01$ and $^{\#\#\#}$ $P < 0.001$ compared to untreated group. * $P < 0.05$, ** $P < 0.01$ and *** $P < 0.001$, compared to the LPS group.

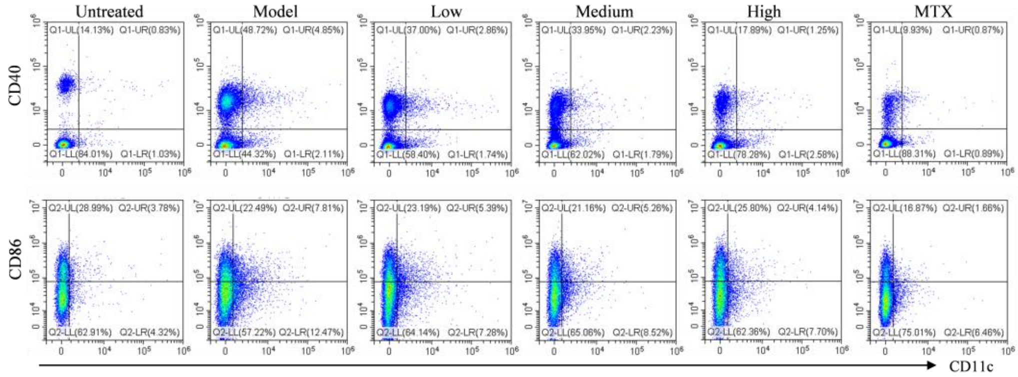

The original dot plot figures in each group of flow cytometry in Figure 4(A).

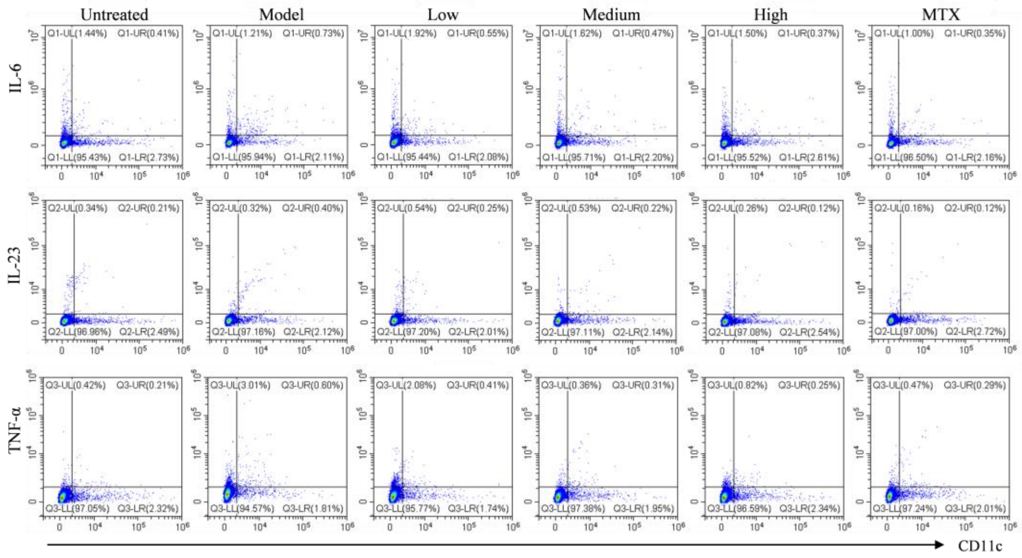

The original dot plot figures in each group of flow cytometry in Figure 4(B).

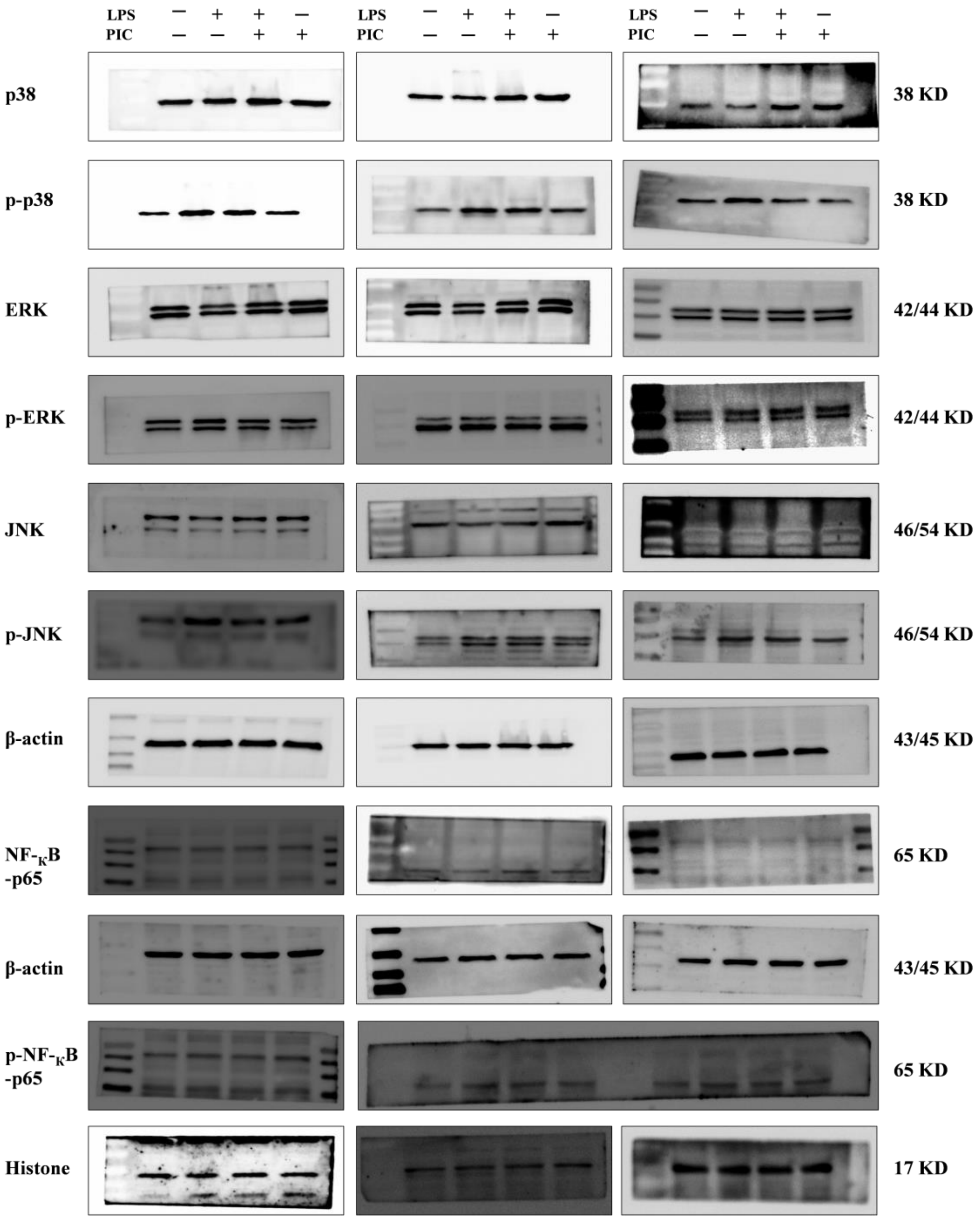

The whole uncropped images of the original Western Blots in Figure 6H.
